# Quantitative polarization-sensitive super-resolution solid immersion microscopy reveals biological tissues’ birefringence in the terahertz range

**DOI:** 10.1038/s41598-023-43857-6

**Published:** 2023-10-03

**Authors:** N. V. Chernomyrdin, D. R. Il’enkova, V. A. Zhelnov, A. I. Alekseeva, A. A. Gavdush, G. R. Musina, P. V. Nikitin, A. S. Kucheryavenko, I. N. Dolganova, I. E. Spektor, V. V. Tuchin, K. I. Zaytsev

**Affiliations:** 1 Prokhorov General Physics Institute of the Russian Academy of Sciences, Moscow, Russia 119991; 2 Research Institute of Human Morphology, Moscow, Russia 117418; 3 Department of Biomedical Engineering, University of Houston, Houston, TX USA; 4 Department of Integrative Physiology, Baylor College of Medicine, Houston, TX USA; 5 Institute of Solid State Physics of the Russian Academy of Sciences, Chernogolovka, Russia; 6 Institute of Physics and Science Medical Center, Saratov State University, Saratov, Russia 410012; 7 Laboratory of Laser Molecular Imaging and Machine Learning, Tomsk State University, Tomsk, Russia 634050

**Keywords:** Polarization microscopy, Terahertz optics

## Abstract

Terahertz (THz) technology offers a variety of applications in label-free medical diagnosis and therapy, majority of which rely on the effective medium theory that assumes biological tissues to be optically isotropic and homogeneous at the scale posed by the THz wavelengths. Meanwhile, most recent research discovered mesoscale ($\sim \lambda$) heterogeneities of tissues; $\lambda$ is a wavelength. This posed a problem of studying the related scattering and polarization effects of THz-wave–tissue interactions, while there is still a lack of appropriate tools and instruments for such studies. To address this challenge, in this paper, quantitative polarization-sensitive reflection-mode THz solid immersion (SI) microscope is developed, that comprises a silicon hemisphere-based SI lens, metal-wire-grid polarizer and analyzer, a continuous-wave 0.6 THz ($\lambda = 500$ µm) backward-wave oscillator (BWO), and a Golay detector. It makes possible the study of local polarization-dependent THz response of mesoscale tissue elements with the resolution as high as $0.15 \lambda$. It is applied to retrieve the refractive index distributions over the freshly-excised rat brain for the two orthogonal linear polarizations of the THz beam, aimed at uncovering the THz birefringence (structural optical anisotropy) of tissues. The most pronounced birefringence is observed for the Corpus callosum, formed by well-oriented and densely-packed axons bridging the cerebral hemispheres. The observed results are verified by the THz pulsed spectroscopy of the porcine brain, which confirms higher refractive index of the Corpus callosum when the THz beam is polarized along axons. Our findings highlight a potential of the quantitative polarization THz microscopy in biophotonics and medical imaging.

## Introduction

During the past decades, various modalities of THz spectroscopy and imaging have been rapidly developed^1,2^. Nowadays, they offer a variety of applications in different branches of science and technology^3,4^. Among them, we particularly notice label-free medical diagnosis of neoplasms^5,6^, diabetic foot syndrome^7^, traumatic injuries^8^, burn wounds^9^, viability^10^, and hydration state^11^ of tissues, monitoring of scar healing^12^ and transdermal drug delivery^13^, therapy of cancers and inflammatory diseases^14,15^. The majority of these applications rely on the effective medium theory and assume biological tissues to be optically isotropic and homogeneous at the THz-wavelength scale^5,16^. The THz response of such tissues is described by relaxation models of complex dielectric permittivity^17,18^ and mostly determined by the content and state of tissue water^3,19^. However, the most recent research revealed mesoscale ($\sim \lambda$) heterogeneities of tissues^3,5,16^ and, thus, posed a problem of studying the THz-wave scattering in such tissues and related polarization effects. Let us consider a few examples.

In Refs.^20–23^, polarization-sensitive diffraction-limited THz imaging was applied to study cancers of the skin and colon, aimed at uncovering changes in the THz-beam polarization upon its interactions with tissues. Despite the observed depolarization of a linearly-polarized incident THz field provided valuable information for the differentiation between intact tissue and cancers, this effect cannot be described within the aforementioned effective medium theory. Due to the diffraction-limited resolution, such THz polarimetry modalities do not provide detailed information regarding the origin of the observed THz-light depolarization (i.e., about any sub-wavelength and mesoscale scatterers in tissues). In Refs.^24,25^, the innovative multi-configuration THz ellipsometry was developed to improve capabilities of the skin tissue characterization via the diffraction-limited THz pulsed spectroscopy. Together with a comprehensive model of the THz-wave–skin interactions, it makes possible quantification of the THz response of both the Stratum corneum (upper layer) and epidermis of the skin. Particularly, it revealed the THz birefringence of the Stratum corneum caused by its structure. While this method greatly enriched the measured THz data, it also obeys the Abbe diffraction limit and does not allow for imaging of separate scatterers in tissues.

In Refs.^26–32^, various modalities of the superresolution THz near-field microscopy were applied for imaging of subwavelength structural elements in biological tissues, such as separate cells. Particularly, in our earlier research, we developed the quantitative $0.15\lambda$-resolution THz SI microscope for imaging of soft biological tissues, which offers high energy efficiency due to the absence of any subwavelength probes or apertures in an optical scheme^2,33^, as well as capabilities of studying spatial distributions of optical properties and water content over the surface of an imaged tissues^34^. It was applied to study mesoscale heterogeneities of different tissues: those of the breast, tongue, brain, and pericardium^33–37^. The observed heterogeneities might lead to the Mie scattering of THz-waves and the related polarization phenomena, as analytically predicted in Ref.^5^.

All these results pose problems of studying the THz radiation–heterogeneous tissues interactions and adapting the radiation transfer theory for describing them. Whereby we note a large number of polarization-sensitive systems capable of studying turbid biological tissues in the visible and infrared ranges^38–45^, such instruments are rare in the THz range, which hampers further research and developments in THz biophotonics and medical imaging. To mitigate this difficulty, in this paper, quantitative polarization-sensitive $0.15 \lambda$-resolution THz SI microscope is developed based on free-standing metal-wire-grid polarizer and analyzer, a continuous-wave 0.6 THz BWO emitter, and a Golay detector. This method is applied to quantify distributions of anisotropic THz refractive index over the freshly-excised intact rat brain ex vivo and, thus, to uncover birefringence of brain tissues at THz frequencies. Thus measured anisotropic THz response of tissues holds information about their structure at the THz-wavelength scale, which is useful in both scientific research and medical applications. High THz birefringence is observed for the Corpus callosum—i.e., a bundle of densely-packed axons bridging the cerebral hemispheres, with the THz refractive index higher when the field is polarized along axons. This effect was then confirmed via the THz pulsed spectroscopy of the porcine brain. Our findings highlight a potential of the quantitative polarization-sensitive THz SI microscopy in different branches of optics, biophotonics, and medical imaging.

**Figure 1:**
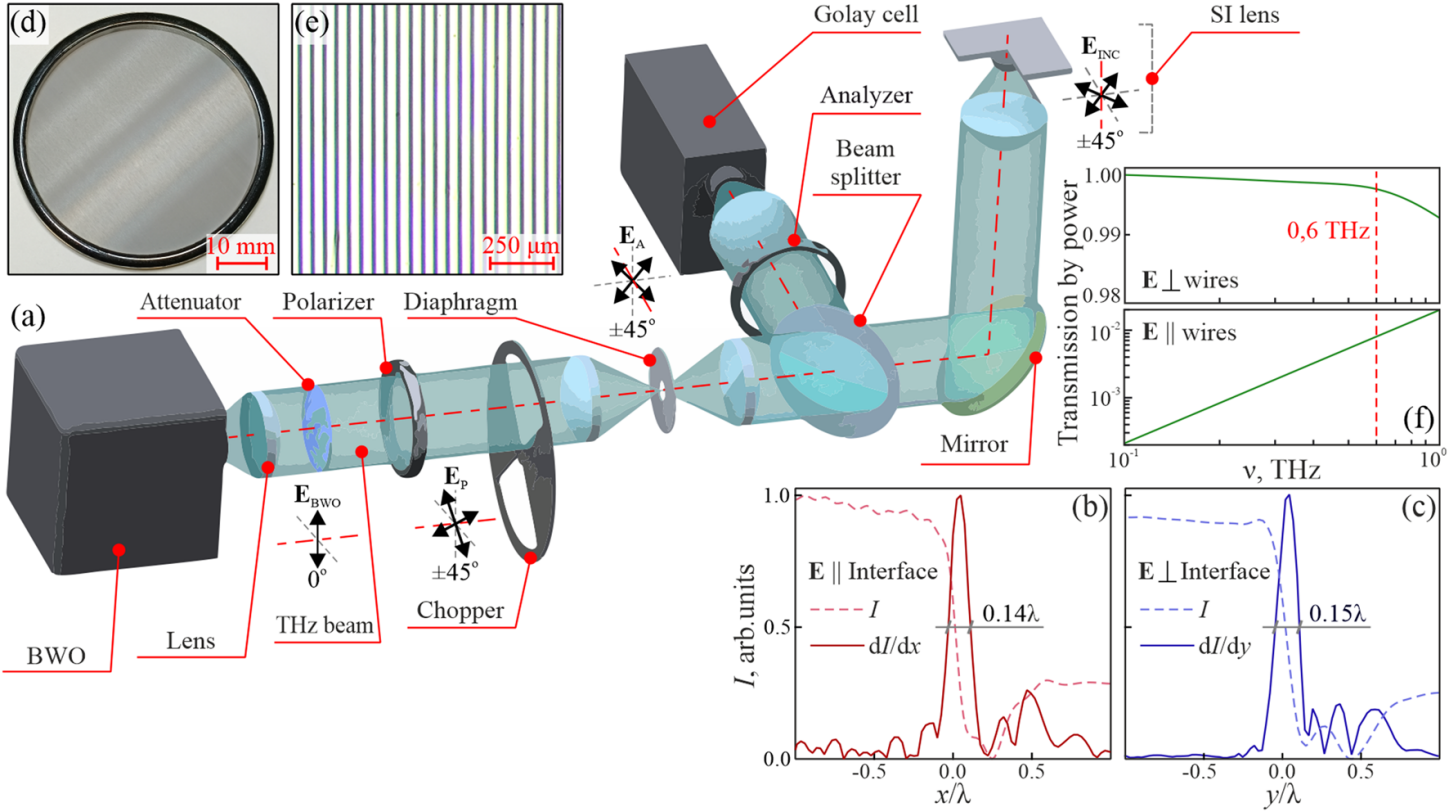
The polarization-sensitive THz SI microscope. (**a**) Schematic of the microscope that uses free-standing metal-wire grid polarizer and analyzer, a continuous-wave 0.6 THz ($\lambda = 500$ µm) BWO emitter, and a Golay detector. (**b**, **c**) Results of the razor blade microscopy that reveal the normalized resolution as high as $\simeq 0.15 \lambda$. (**d**–**f**) Photo, optical microscopy, and transmission spectra of the polarizer.

### Human and animal conflicts

The work with laboratory animals was carried out by Anna I. Alekseeva at the experimental base of the Research Institute of Human Morphology (Moscow, Russia). The latter is fully equipped to conduct such studies. This work was carried out in accordance with the international ethical standards and principles: (i) The European Convention for the Protection of Vertebrate Animals used for Experimental and Other Scientific Purposes (Strasbourg, 2006); (ii) The International Guidelines for Biomedical Research in Animals (CIOMS and ICLAS, 2012); (iii) The 3R principles (replacement of laboratory animals in the experiment with alternative models when possible; reduction in the number of studied animals; refinement of experimental methodology including the pain relief and animal welfare). Also, this research followed the internal rules of the Research Institute of Human Morphology. Only properly qualified personnel are allowed to work with animals in this organization. This allows to fulfill the norms and ethical principles of working with animals. Animals are kept in the vivarium in proper conditions. All procedures with animals are carried out taking into account their condition to ensure the reliability of scientific results.

## Results

### Experimental setup

In Fig. 1a, we show a scheme of the developed polarization-sensitive THz SI microscope, which is quite similar to that detailed in Refs.^33–37^, except the use of polarizer and analyzer. Let us re-iterate that our THz microscopy relies on the in-house BWO, as the continuous-wave emitter at 0.6 THz ($\lambda = 500$ µm), and the in-house Golay cell, as the THz-beam power detector. The BWO beam is modulated at 22 Hz using a mechanical chopper and, then, demodulated via the lock-in principle. A 1 × Kepler telescope, with a sub-wavelength metal aperture at its intermediate focal plane, is used to homogenize the THz beam over its aperture.

The key element of our microscope is the in-house reflection-mode THz SI lens, which comprises a wide-aperture aspherical singlet and a near-focal hemisphere, made of the high-resistivity float-zone silicon (HRFZ-Si) and serving as a resolution enhancer^33^. In turn, this hemisphere comprises a rigidly fixed hypohemisphere and a movable window, atop of which an imaged object is placed. Such a composite construction of the hemisphere makes possible visualizing amorphous objects and soft biological tissues by their raster-scan with a focused THz beam. These two mechanically-independent elements are kept in contact to form a unitary optical one—i.e., the HRFZ-Si hemisphere with the refractive index of $n_\text{Si}=3.415$. A small distance behind this hemisphere, in free space, the sub-wavelengths focal spot is formed. In Fig. 1b and c results of the razor-blade test (i.e., imaging of a metal-dielectric test object with abrupt changes in reflectivity) are shown. They reveal the normalized resolution of our microscope as high as $\simeq 0.15 \lambda$^33^, which depends (to some extent) on the optical properties of an imaged object^34^.

**Figure 2:**
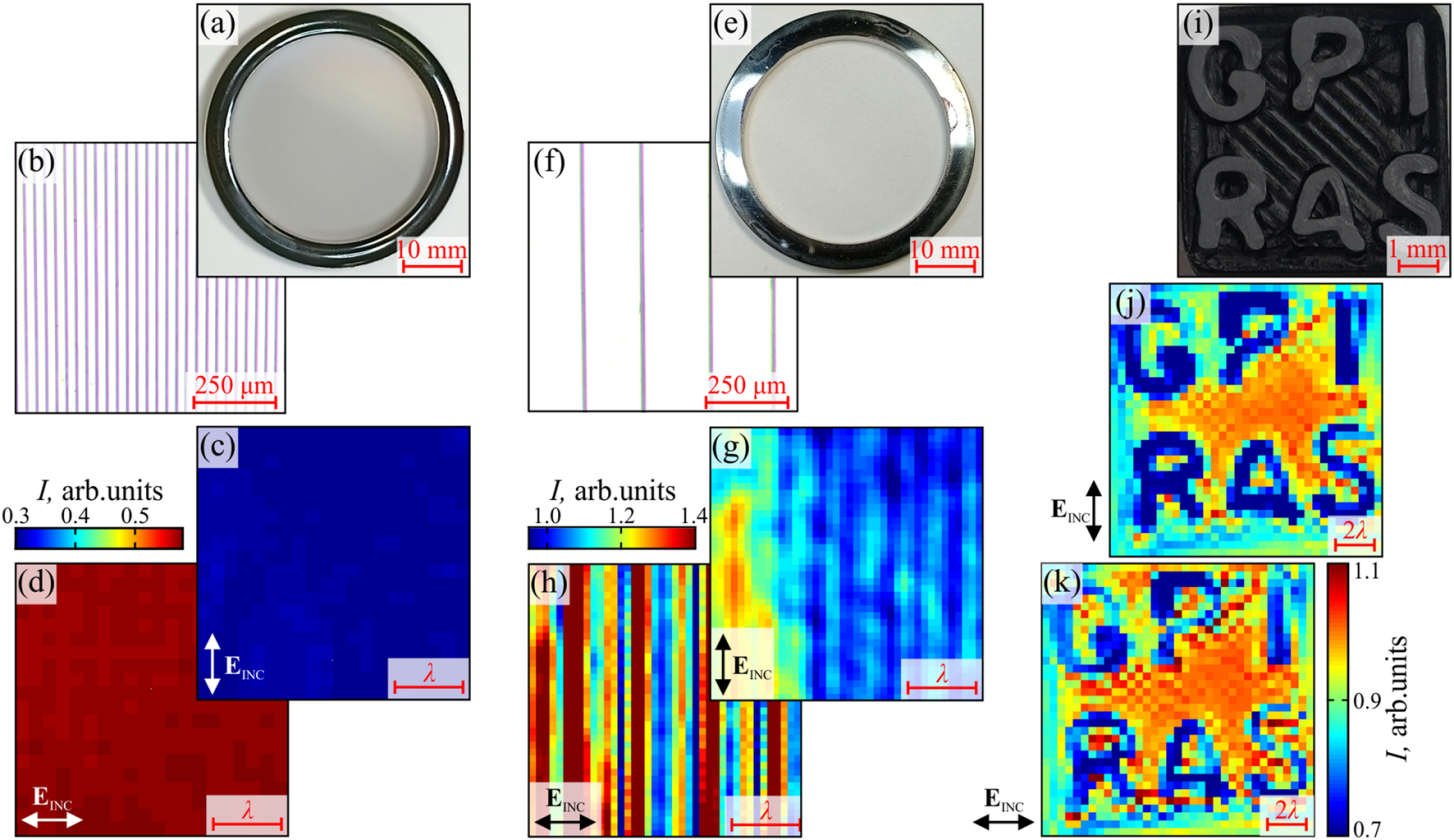
Polarization-sensitive 0.6 THz ($\lambda = 500$ µm) SI microscopy of test objects. (**a**–**d**) Photo, optical microscopy, and THz images, respectively, of a free-standing metal-wire grid with the strongly sub-wavelength period of $\simeq 0.06 \lambda$. In these THz images, individual wires cannot be resolved, while their anisotropic effective THz response is obvious. (**e**–**h**) Equal data for a free-standing metal-wire with the mesoscale period of $\simeq 0.4 \lambda$. In these THz images, individual wires are resolved, and optical anisotropy of THz-wave scattering on wires is evident. (**i**–**k**) Photo and THz images of the 3D printed GPI RAS logo.

Initially, the BWO beam is weakly polarized in the vertical direction ($\textbf{E}_\text{BWO}$), with the degree of polarization as small as $\simeq 0.7$. In Fig. 1d and e, the in-house free-standing metal-wire-grid polarizer is shown, which is formed by an array of tungsten wires with the diameter and period of 15 and 50 µm, respectively. It allows for achieving a linear THz-beam polarization ($\textbf{E}_\text{P}$) with the $\pm 45^\circ$ electric field orientations towards the initial BWO one ($\textbf{E}_\text{BWO}$). Switching between these two orthogonal polarizations is performed via manual rotation of the polarizer. For the $\pm 45^\circ$ polarizations, the intensity of THz beam radiating a sample and, then, reaching the detector is almost equal, since these polarizations suffer mostly equal Fresnel loss while interacting with optical interfaces. This ensures similar signal-to-noise ratios in the collected THz polarization images. In Fig. 1f, we show the transmission spectra (by power) of our polarizer, when the THz field is oriented perpendicularly and collinearly to the metal wires. One can notice high efficiency of the applied polarizer.

From Fig. 1a, one notices that the analyzer is mounted in front of the detector, while its geometry and characteristics are equal to those of the polarizer. The analyzer can be manually rotated, while in this study, it is switched between the $\pm 45^\circ$ polarizations to capture the co-polarized components of the scattered THz field ($\textbf{E}_\text{A}$). In the described microscope arrangement, switching between the $\pm 45^\circ$ polarizations is performed only via rotation of the polarizer and analyzer. This makes it possible to probe an imaged object (placed atop of the HRFZ-Si window) by these two orthogonal polarizations without any object’s displacements and rotations. This is of crucial importance when visualizing soft biological tissues, because it eliminates any shift and shrinkage of tissues and, thus, the related THz image distortions.

It is worth noting that, despite the described THz SI microscope operates at the particular BWO frequency of 0.6 THz ($\lambda = 500$ µm), our SI lens and polarizer/analyzer are capable of operation at other THz frequencies or even of broadband operation. In this way, our THz microscope can be combined with other continuous-wave monochromatic or broadband pulsed THz emitters, aimed at accommodating the needs of different THz applications.

### THz microscopy of test objects

The developed THz microscope is applied for imaging of few test objects. Among them, we first consider the sub-wavelength and mesoscale arrays of tungsten wires, similar to those used in the described polarizer and analyzer.

In Fig. 2a and b, we show photo and optical microscopy of the sub-wavelength metal-wire array with the period of 30 µm ($0.06\lambda$) and the wire diameter of 15 µm, while in Fig. 2c and d, THz images of this array placed atop of the HRFZ-Si window are shown for the incident THz field ($\textbf{E}_\text{INC}$) polarized along and transverse the wires, respectively. Since the wire period ($0.06\lambda$) is much smaller than the microscope resolution ($0.15 \lambda$), individual wires are not resolved in our THz images. However, these images reveal higher intensity of the THz field back-scattered by this array, when the incident field $\textbf{E}_{\text{INC}}$ is polarized transversely. This effect can be explained by the anisotropic optical response of a wire medium. When a simple wire medium is suspended in free space, the tensor of its complex dielectric permittivity takes the form^46^:

1 $$\begin{aligned} \mathbf {\varepsilon } = \left( \begin{matrix} \varepsilon _{x} &{} 0 &{} 0 \\ 0 &{} \varepsilon _\text{t} &{} 0 \\ 0 &{} 0 &{} \varepsilon _\text{t} \end{matrix} \right) , \qquad \varepsilon _{x} = 1 - \frac{k_\text{p}^{2}}{k_\text{0}^{2}}. \end{aligned}$$

Here, the *x* axis is parallel to the wires, $\varepsilon _{x}$ and $\varepsilon _\text{t}$ stand for the axial and transverse component of the dielectric permittivity tensor; $k_\text{p}=\omega _\text{p} / c_\text{0}$ is the wavenumber corresponding to the effective plasma frequency $\omega _\text{p}$ that depends on the wire diameter, the lattice type and period; $k_\text{0}$ is the wavenumber in free space, and $c_\text{0} = 3 \times 10^8$ m/s is the speed of light in free space. Usually, the axial dielectric permittivity is similar to that of a collisionless non-magnetized plasma (described by the Drude model) and differs from unity ($\varepsilon _\text{x} \ne 1$). Once the volume fraction ratio of wires is small, the orthogonal dielectric permittivity turns into unity ($\varepsilon _\text{t} = 1$). At frequencies below $k_\text{p}$, Eq. (1) represents a wire medium as an indefinite dielectric metamaterial with $\mathfrak {Re} \left( \varepsilon _\text{x} \right) < 0$ and $\mathfrak {Re} \left( \varepsilon _\text{t} \right) > 0$^46^. Such an effective optical anisotropy of a wire medium gives qualitative description of the observed differences in the THz polarization images from Fig. 2c and d, while further quantification of this effect require additional studies.

In Fig. 2e and f, we show photo and optical microscopy of mesoscale metal-wire array with the period of 200 µm ($0.4\lambda$) and the wire diameter of 15 µm, while in Fig. 2g and h, the corresponding THz images are presented. Since the wire period in this array ($0.4\lambda$) exceeds the resolution of our system ($0.15\lambda$), individual wires are clearly resolved in our THz images, also distorted by a speckle pattern. One notice high intensity of the THz field back-scattered on the individual wires when it is polarized trasnversely. This effect can be explained by the different total scattering cross-sections $\sigma _{\parallel }$ and $\sigma _{\perp }$ of a plane wave on a thin cylindrical metal object in a Rayleigh limit, when the wave is polarized collinearely and perpendicularely to the cylinder^47^:

2 $$\begin{aligned} \sigma _{\parallel } = - \frac{ \pi ^{2} a^3 k_\text{0}}{2}\mathfrak {Im}\left[ \varepsilon _\text{m} - 1 \right] , \qquad \sigma _{\perp } = - \pi ^{2} a^{3} k_\text{0} \mathfrak {Im} \left[ \frac{ \varepsilon _\text{m} - 1 }{ \varepsilon _\text{m} + 1 } \right] . \end{aligned}$$

Here, *a* is a cylinder radius ($a \ll \lambda$), and $\varepsilon _\text{m}$ is a complex dielectric permittivity of bulk metal defined by the Drude model.

Next, in Fig. 2i we show a photo of the GPI RAS logo, that is 3D printed (FlashForge Creator Pro 2) of a conductive compound of acrylonitrile butadiene styrene (ABS) polymer and carbon nanotubes with the resistivity of $4.64\times 10^2$ $\Omega$ cm. The total lateral dimensions of the logo are 7 × 7 mm^2^, while the depth and width of letters are both 0.4 mm, being limited by the size of a 3D printer’s nozzle. In Fig. 2j and k, polarization THz images of this logo reveal noticeable anisotropy, that might be attributed to the THz-wave scattering at the object’s edges, as well as birefringence of the ABS material caused by its patterning during the 3D printing.

Thereby, the polarization-sensitive THz SI microscopy of the test objects highlight a potential of this novel imaging modality in studies of the anosotropic THz response of various media with an essentially sub-wavelength resolution.

### THz microscopy of the rat brain

Structural optical anisotropy (birefringence) is a common effect that was earlier observed in the visible and infrared ranges for a variety of fibrous tissues formed by well-aligned, densely-packed, and oriented cylindrical/elliptical scatterers^48^. Particularly, in the infrared range, this effect was found in the Corpus callosum of the brain, formed by bundles of axons bridging the cerebral hemispheres^49^. Meanwhile, in the THz range, this effect is rarely studied: namely, it was observed only for the *Stratum corneum* of the skin via the THz ellipsometry^24^. To uncover tissue birefringence in the THz range at the sub-wavelength scale, the developed THz microscope is applied to study ex vivo the freshly-excised intact rat brain, with a focus on the Corpus callosum.

**Figure 3:**
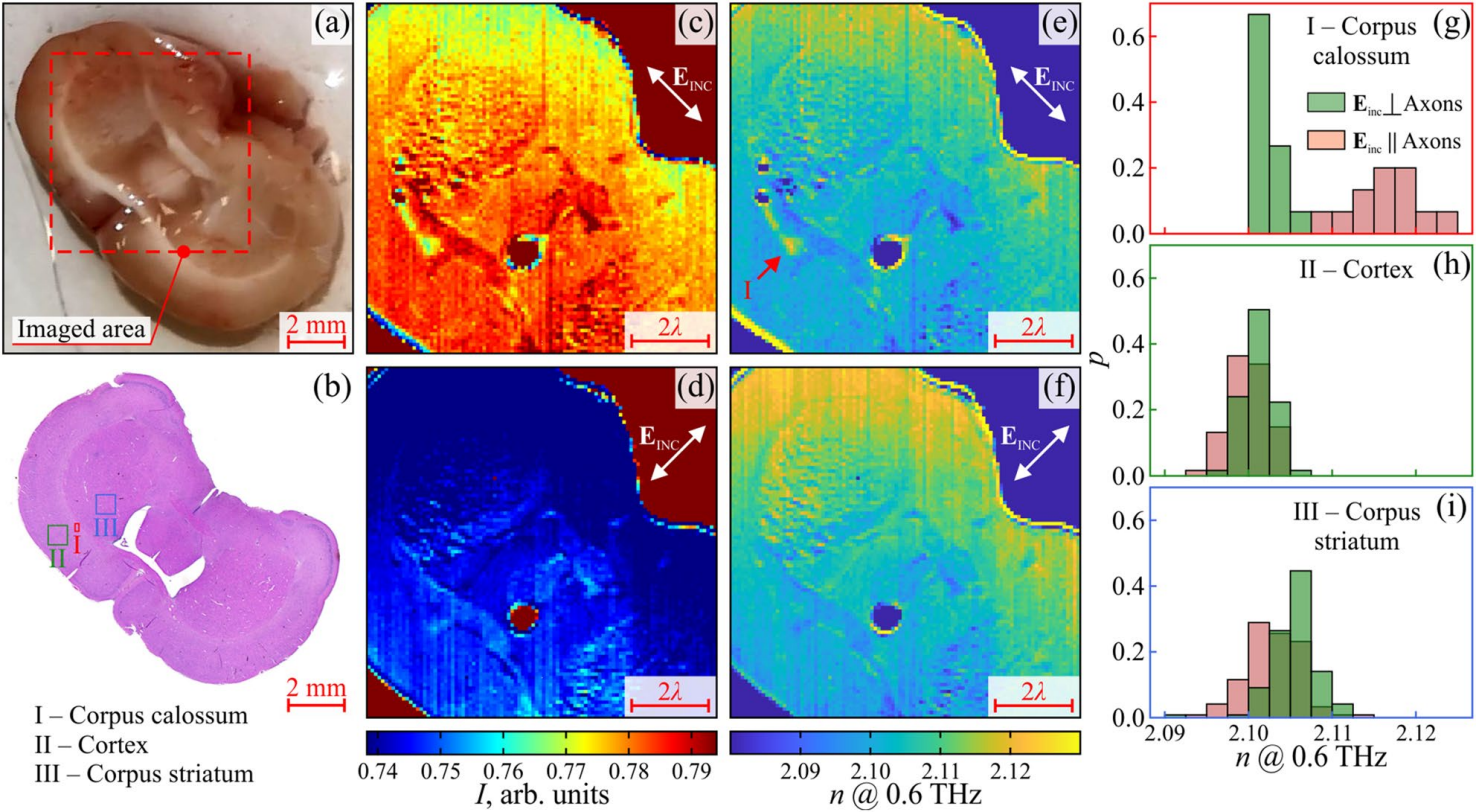
Polarization-sensitive 0.6 THz ($\lambda = 500$ µm) SI microscopy of the rat brain ex vivo. (**a**, **b**) Photo and H&E-stained histology of the brain, where an imaged area is pointed out, along with three areas of interest—i.e., the Corpus callosum (I), Cortex (II), and Corpus striatum (III). (**c**, **d**) THz images of the rat brain for the two orthogonal orientations of the THz-beam polarization $\textbf{E}_{\text{INC}}$ in the imaging plane, which are collinear and transverse to axons in the Corpus callosum. (**e**, **f**) THz refractive index distributions *n* over the imaging area for the polarizations. (**g**–**i**) Histograms for the THz refractive indices of the brain areas I, II, and III, respectively, where (**g**) shows significant THz birefringence of the Corpus callosum, when the THz field $\textbf{E}_{\text{INC}}$ is collinear and transverse to axons.

The Wistar rat is sacrificed by carbon dioxide asphyxiation. The cranium is opened to remove the whole rat brain. The scalpel incision is made in the frontal plane approximately through the Corpus callosum. The dissected fragment is placed atop the HRFZ-Si window of our microscope (Fig. 1a) in a way that the incision side is in contact with the window, while its back side is covered by a thin polyethylene film (it preserves tissues for hydration / dehydration and sustains their THz response unaltered during measurements). After the THz microscopy, the tissue specimen is fixed (for 48 h) in a 4% paraformaldehyde solution and subsequently embedded into a paraffin block. Hematoxylin and Eosin (H&E) staining of 5-µm-thick tissue sections is performed, while their histological examination makes possible identifying and marking brain structures. In Fig. 3a and b, we show photo of the freshly-excised intact brain, and the related H&E-stained histology.

During the THz measurements, the incident THz beam polarization ($\textbf{E}_\text{INC}$) is, first, set to be collinear and, then, transverse to the bundle of axons in the Corpus callosum (those lie in the imaging plane). The resultant THz images of the $10\times 10$ mm^2^ tissue area are shown in Fig. 3c and d. By resolving the inverse problem of THz SI microscopy as detailed in Ref.^34^, in Fig. 3e and d, we quantify distributions (over the imaging area) of the tissue refractive index *n* at 0.6 THz for the two orthogonal polarizations. Our THz microscope allows for studying the response of only the superficial tissues, which is due to a limited depth of field^33^ and a small depth of THz-wave penetration in tissues^18^; both are $\sim 100$ µm. One can clearly observe differences between the THz images and *n*-distributions for the two orthogonal polarizations. In the H&E-stained histology (Fig. 3b), three areas of interest are pointed out: (I) the Corpus callosum, (II) Cortex, and (III) Corpus striatum. For these areas, normalized statistical distributions of the tissue refractive index $p \left( n \right)$ are shown in Fig. 3g–i. As expected, in (g), quite high birefringence is evident for the Corpus callosum (I), where *n* is higher for the THz field $\textbf{E}_\text{INC}$ polarized along axons. In turn, in (h) and (i), the Cortex (II) and the Corpus striatum (III) show almost no birefringence due to their isotropic morphology at the THz-wavelength scale. In Fig. 3e and f, less pronounced anisotropy is observed for other areas of the brain, including the white matter and the gray matter, but the detailed analysis of their THz response is postponed to our future work.

### THz spectroscopy of the porcine brain

To verify the anisotropic response of the Corpus callosum from rat brain uncovered by the THz microscopy, we now move to studying that from the porcine brain ex vivo using the THz pulsed spectroscopy. The selection of the porcine brain is determined by the dimensions of the Corpus callosum, which are large enough to be characterized by the diffraction-limited THz spectrometer. The porcine brain is procured and meticulously handled following approved guidelines and standards. The Corpus callosum tissues are fixated (using a Harvard Apparatus Programmable Syringe Pump system, USA), and carefully dissected. The supplying blood vessels are cannulated with appropriate-sized needles. The automated pump is programmed to deliver a 10% neutral buffered formalin solution at a controlled flow rate. The perfusion is performed for 2 h to ensure uniform fixation of tissues. Visual monitoring and adjustment of the flow rate during the perfusion process allow to maintain consistent perfusion pressure. Following fixation, tissues are processed for downstream applications, such as sectioning and histological analysis. To prepare the tissue sample, the brain is sectioned via a cryotome (Sakura, USA). A delay between the preparation and THz spectroscopy of tissues is no longer than 2 h, which ensures their freshness and minimize any potential changes in their biochemistry or morphology that can interfere with the experiment. In this way, the sections of a uniform thickness $\simeq 200$ µm are prepared for the THz spectroscopy.

**Figure 4:**
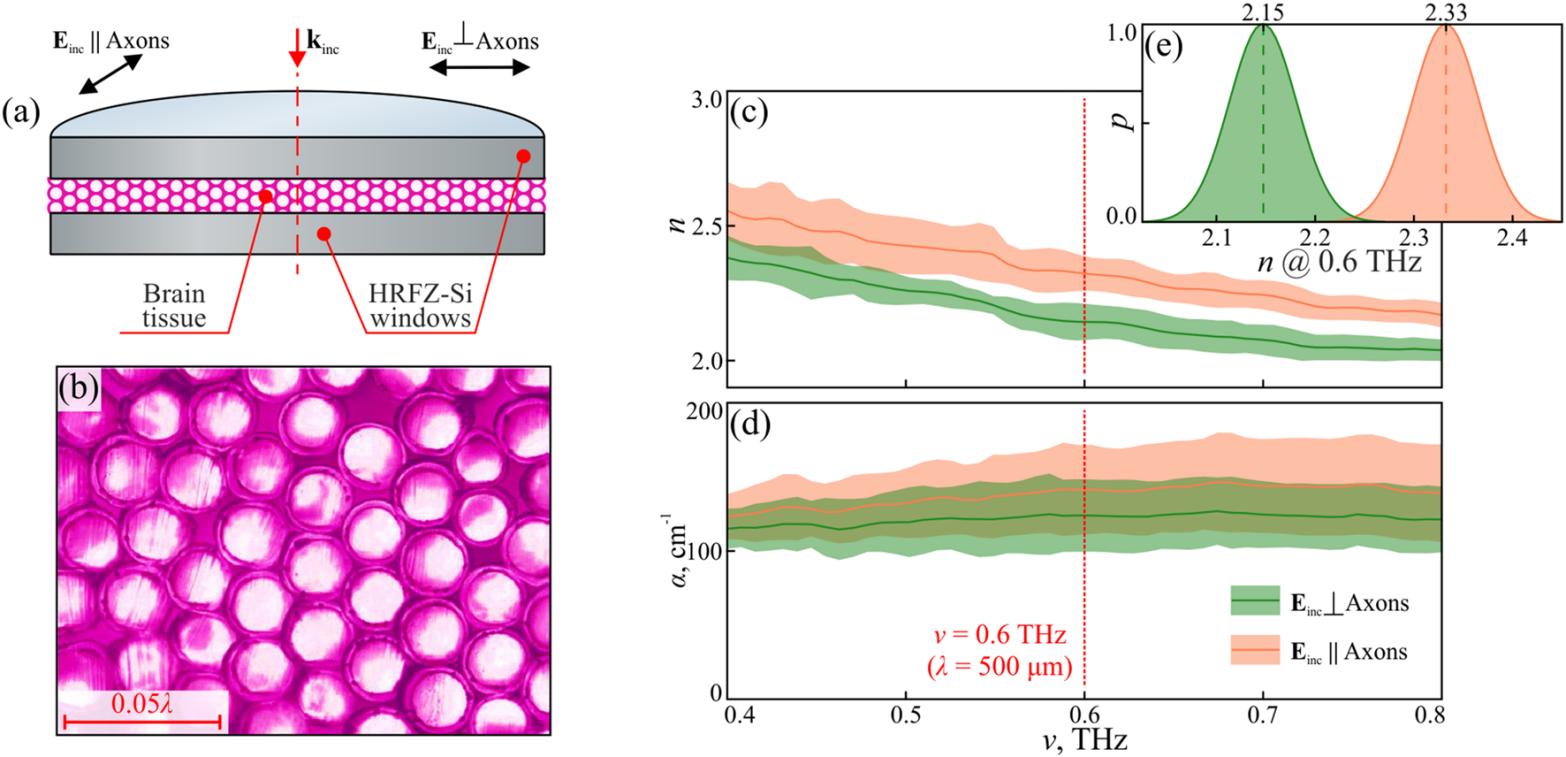
THz pulsed spectroscopy ex vivo of the Corpus callosum from the porcine brain ex vivo. (**a**) Schematic of the THz measurements of brain tissues, sandwiched between the two HRFZ-Si windows of a cuvette. Here axons are perpendicular to the THz beam axis. (**b**) H&E-stained histology of the Corpus callosum from the porcine brain, with axons packed in an almost perfect hexagonal lattice. (**c**, **d**) THz refractive index *n* and absorption coefficient $\alpha$ (by field) of the Corpus callosum, for the THz field $\textbf{E}$ oriented collinearly and transversely to axons. The error bars represent the data reproducibility. (**e**) Gaussian fits for the refractive index *n* at 0.6 THz of the Corpus callosum at the two polarizations.

In this study, the in-house transmission-mode THz pulsed spectrometer is used, that was detailed in Ref.^50^. It uses a pair of photoconductive antennas, as a emitter and a detector of THz pulses, and the in-house metal-wire-grid polarizer (see Fig. 1d–f) to probe tissues with a linearly-polarized THz beam. During measurements, the Corpus callosum from the porcine brain is sandwiched between the two thick HRFZ-Si windows of a cuvette (also detailed in Ref.^50^), where bundles of axons are orthogonal to the optical axis (Fig. 4a). The cuvette with tissues is introduced into the THz beam path, the THz beam is focused on the cuvette, and then collimated by a pair of equal off-axis parabolic mirrors, while the THz focal spot is smaller than the sample aperture^50^. By rotating the cuvette around the THz optical axis, the tissue transmission is measured when the THz beam is polarized collinearly and transversely to axons. Then, THz optical properties of tissues are retrieved in the 0.4–0.8 THz range, as detailed in Ref.^50^.

For histology of the porcine brain tissues, a modified H&E-staining protocol is used, aimed at preserving axon myelin. After fixation, the tissue sections are dehydrated through a graded series of ethanol solutions. To avoid excessive extraction of lipids and myelin components, the dehydration step is controlled to preserve the myelin sheath. Then, the sections are incubated in a modified hematoxylin solution with a reduced concentration of acidic alcohol, preventing overstaining and damage to the myelin structure. Counterstaining with eosin is performed using a brief incubation period to limit the penetration of eosin into the myelin sheath. This modified staining procedure aimed to achieve a balanced contrast between cellular components and myelin, allowing for accurate visualization and analysis of axon morphology and myelin integrity. In Fig. 4b, H&E-stained histology is shown for the transverse cross-section of the Corpus callosum, where axons form almost perfect hexagonal lattice.

In Fig. 4c and d, the refractive index *n* and absorption coefficient $\alpha$ are retrieved from the THz pulsed spectroscopy data for the Corpus callosum of the porcine brain, where green and orange curves correspond to the THz field polarized transversely and collinearly to axons, while the shaded areas—the $\pm 2\sigma$ confidence intervals. Notable variability of the measured THz refractive index and absorption coefficient of the brain can be attributed to the following factors:

- Our THz pulsed spectrometer features the diffraction-limited resolution, which is about 5 mm (at the object plane) at lower THz frequencies for the particular aperture of an optical system. The measured THz response of tissues is averaged within this focal spot, while all unresolved mesoscale or sub-wavelength heterogeneities of tissues might lead to some fluctuations of the measured data.
- THz pulsed spectroscopy of tissue is carried out at an ambient atmosphere, without vacuumization or nitrogen gas purging of the THz beam path. Fluctuations of humidity inside the THz chamber can lead to some changes in the water vapor content inside it and, thus, instability of the THz beam intensity passing it during quite a long-term experiment.
- When retrieving the THz pulsed spectroscopy data, the measured THz spectra of tissues are averaged over 5 independent measurements within the $\pm 5^\circ$ sample rotation angle. This also results in the measured THz data variability.

In Fig. 4c and d, quite monotonic frequency-dependent changes of the measured THz optical properties of porcine brain are notable, which are common for any hydrated tissues in the THz range. In fact, such THz response is mostly determined by the relaxation-like dynamics of tissues water with its very broad absorption bands^3^.

In Fig. 4e, Gaussian fits for the *n*-values at 0.6 THz are shown. A notable birefringence is observed from the THz spectroscopy data in complete accordance with the THz microscopy from Fig. 3g. Indeed, THz spectroscopy also reveals higher refractive indices *n* of the Corpus callosum, when for the THz field is polarized collinearly to axons, thus, justifying correctness of our THz microscopic measurements. Meanwhile, THz spectroscopy uncovers overall higher *n*-values of brain tissues, along with larger contrast for the two orthogonal polarizations. Such differences can be attributed to a number of factors: tissues from different model animals are studied by microscopy and spectroscopy; slightly distinct protocols are used for preserving rat and porcine brain tissues; cross-sections of the brain considered in the discussed two experiments also differ due to small dimensions of the Corpus callosum (especially in the rat brain) and, thus, a delicate procedure of these tissue incision; reconstruction of the THz optical properties of tissues in microscopy^34^ and spectroscopy^50^ involve different methods with their own drawbacks and limitations.

The reported THz birefringence of the Corpus callosum might originate owing to the two main factors. First, different Rayleigh scattering of THz waves, with the two orthogonal polarizations, on axons, as cylindrical dielectric particles^47^, is expected. Second, axons are filled with aqueous solutions of electrolytes, which can lead to their anisotropic Drude-like conductivity [similarly to the aforementioned wire medium^46^; see Eq. (1)], but this factor should be less-pronounced due to the mostly relaxation-like THz complex dielectric permittivity of brain tissues determined mostly by the content and state of tissue water^18^. At this moment, it is hard to quantify an interplay between these factors, therefore, more investigations are in order.

## Discussions

Despite the demonstrated sensitivity of THz waves to the local ordering and orientation of axon bundles in the white matter of the brain, we should stress that, for such a demonstration, the Corpus callosum was intentionally oriented, so that axons were, first, collinear and, then, transverse to the incident linearly-polarized THz field. Generally, to study the axons’ packing in the white matter, one needs to resort to probing tissues with a larger number of polarizations, under different tissue orientations and incidence angles. Cross-polarized component of the scattered THz field can become a source of the additional useful information^21^, along with the analyzed co-polarized one. After such an increase in the information content, the polarization-sensitive THz SI microscopy can be considered as a prospective tool of cognitomics, aimed at studying axons packing in the white matter of the brain. THz birefringence might be inherent to a variety of biological tissues, including those from humans or animals (such as fibrous tissues of the muscle and tendon, capillaries and veins)^33,35^, or even those from plants (veins and capillaries)^33,51^. Thereby, besides the aforementioned applications in cognitomics, the polarization-sensitive THz SI microscopy holds a potential in other branches of medical imaging, including applications in ophthalmology (studying the cornea pathologies^52^), oncodiagnosis (diagnosis of melanoma^53^, basal cell carcinoma^54^, lung cancer^55^, brain tumors^16^, etc.), cardiovascular diseases (study disorders of the bladder wall^56^, or diabetic foot^7^), and others.

The polarization-sensitive THz SI microscopy can be further aided by the Mueller matrix formalism^57,58^, that employs a 4 × 4-matrix for describing transformation of the electromagnetic-wave polarization upon its interaction with a turbid medium. Decomposition of the Muller matrix gives a reasonable estimate for such polarization parameters of a turbid medium, as optical rotation and linear retardance^59,60^. Different instrumental realizations of the polarization imaging have been proposed in the visible and infrared ranges, including the classic approach (it requires 36 measurements with different combinations of polarizer and analyzer to estimate the 4 × 4 Mueller matrix), or the rotating retarder approach (it reduces the number of measurements down to 24)^61^. Considering such a large number of images to be acquired by the THz microscope for the Mueller matrix analysis, we conclude the need for boosting the performance of our system, improving its operation rate, and sensitivity.

- First, we should optimize acquisition time of our THz SI microscope by exploiting faster pyroelectric detector and raster scanning system utilizing fast stepper motors. This would allow us to reduce single scan time down to 2–3 min, which is important for the Mueller matrix polarimetry, that requires tens of polarimetric images to be collected.
- Second, estimation of the Mueller matrix demands not only linear, but also elliptical polarizations of the incident THz beam. To realize such a set of linear and elliptical THz-beam polarizations, one can either use classical polarization converters based on the THz birefringent materials (crystalline quartz, sapphire, etc.), or resort to emerging approaches based on distinct physical effects in graphene^62^, vanadium dioxide^63^, metal-wire grating^64^, multilayer metamaterials^65^, 3D printed conductive polymer structures^66^, and parallel-plate splitters^67^.
- Third, in the Muller matrix polarimetry, one usually considers interaction between low-aperture beams and a sample, while in the THz SI microscope, one deals with a very wide aperture and excitation of evanescent waves at the SI lens–object interface. To combine the Muller matrix polarimetry with the THz SI microscopy, one should account for such a wide beam aperture and, thus, modify the underlying physical models.

We should stress the benefits of implementing polarization measurements in the THz range, as compared to others. First, the studied spectral features of matter (absorption lines or bands) differ for various spectral ranges due to the different quantum energy and, thus, distinct mechanisms of the electromagnetic-wave–matter interactions. Second, the studied polarization effects are associated with electromagnetic-wave scattering and, thus, determined by a ratio between the wavelength and dimensions/shapes of the object’s structural elements. In this way, polarization measurements in different spectral ranges (including THz) probe various scales of the object’s heterogeneity. Therefore, we conclude that THz spectroscopy and microscopy hold specific information about the imaged objects, that somewhat complement spectroscopic and imaging data from other ranges. Generally, to better understand the electromagnetic-wave–object interactions and to select optimal spectral range for addressing some particular applied problem (such as non-destructive quality control or medical diagnosis), it is better to, first, perform broadband measurements and, then, select some particular optimal spectral bands and wavelengths.

Reflection- or transmission-mode polarization imaging, or its fiber-based endoscopic realization^44^, are available in the visible and infrared ranges. All these techniques can be translated to the THz range in order to shine the light on the polarization effects of the THz-wave–tissue interaction. Extending the information content of the polarization-sensitive THz SI microscopy, developing novel technique to collect and analyze the data, as well as exploring their biomedical applications form a promising research avenue.

## Conclusions

In this paper, quantitative continuous-wave polarization-sensitive $0.15 \lambda$-resolution 0.6 THz SI microscope was developed and applied to study spatial distributions of the anisotropic THz optical properties of test objects and brain tissues. Capabilities of mapping, at a sub-wavelength scale, the anisotropic THz response of different objects using the developed THz imaging modality were objectively demonstrated. This allowed us to uncover the THz birefringence of freshly-excised intact rat brain ex vivo, with the most pronounces anisotropy inherent to the *Corpus callosum*—a bundle of well-aligned, densely-packed, and oriented axons connecting the cerebral hemispheres. Our findings emphasize a potential of the quantitative polarization-sensitive THz SI microscopy in biophotonics and medical imaging.

## Data Availability

Data underlying the results of this paper are not publicly available at this time, but may be obtained from the corresponding authors NVCh (chernik-a@yandex.ru) and KIZ (kirzay@gmail.com) upon reasonable request.
